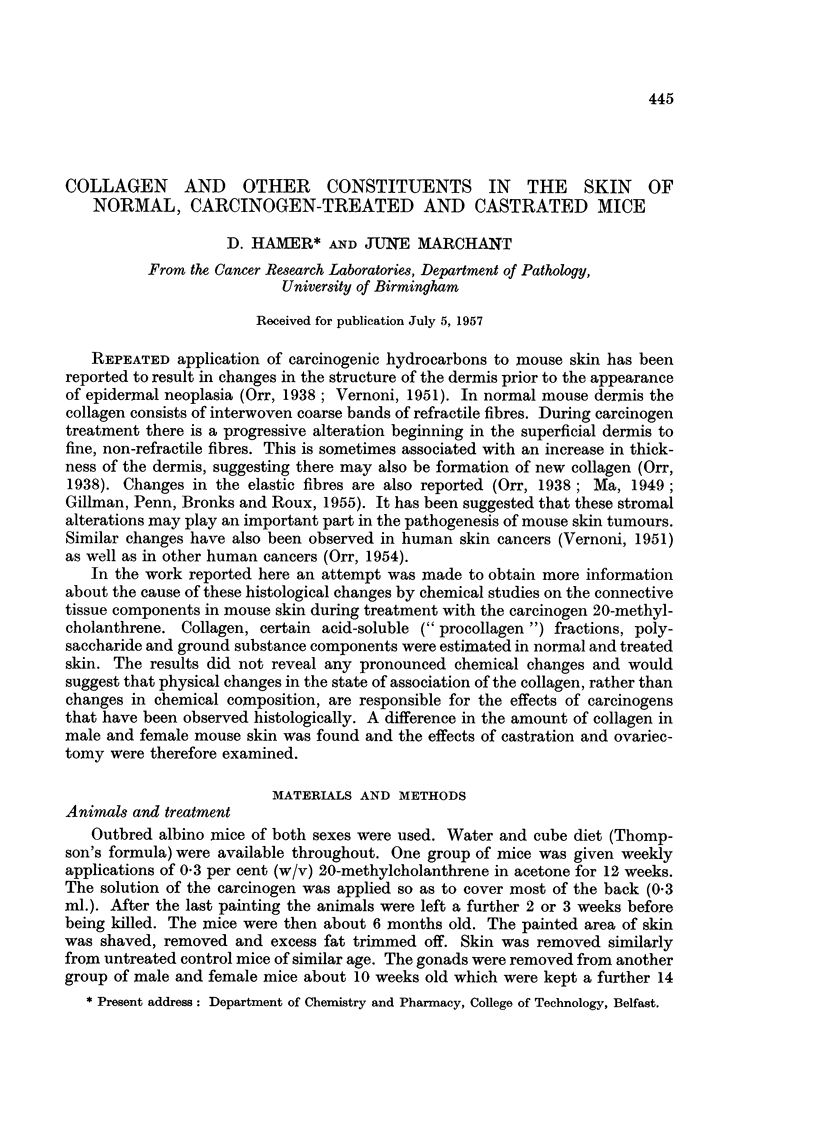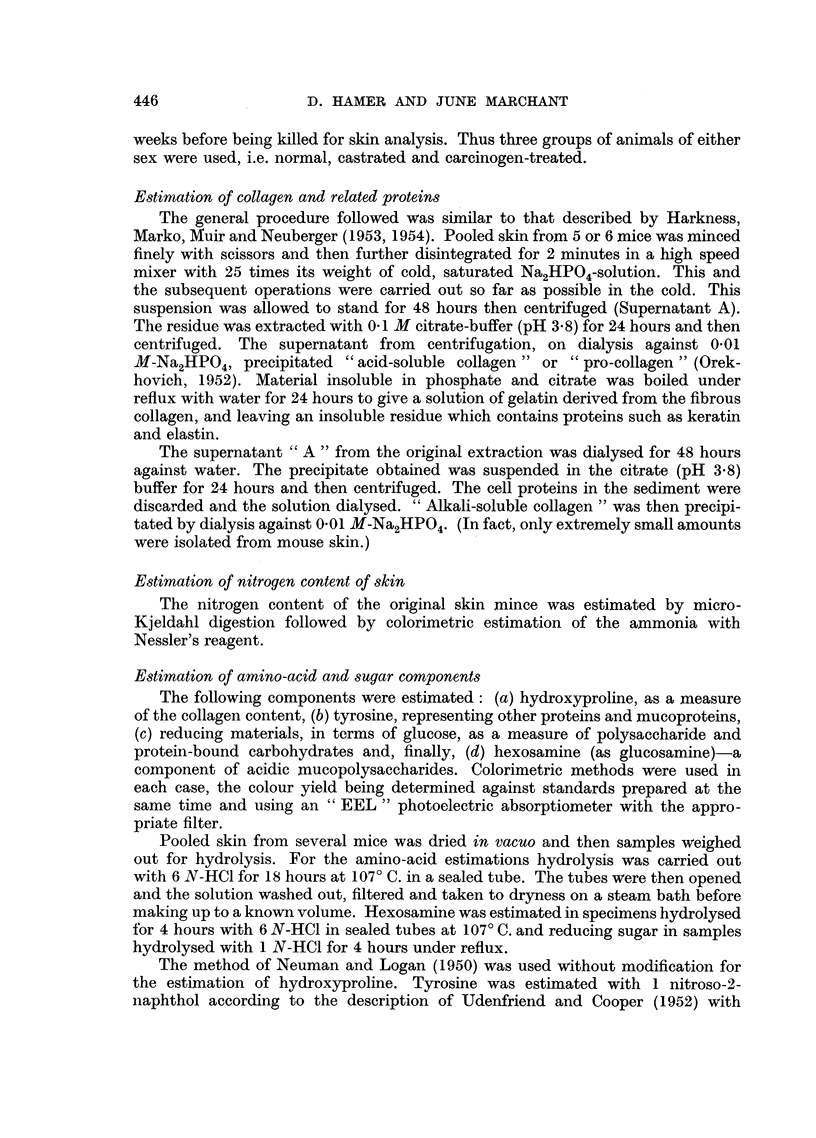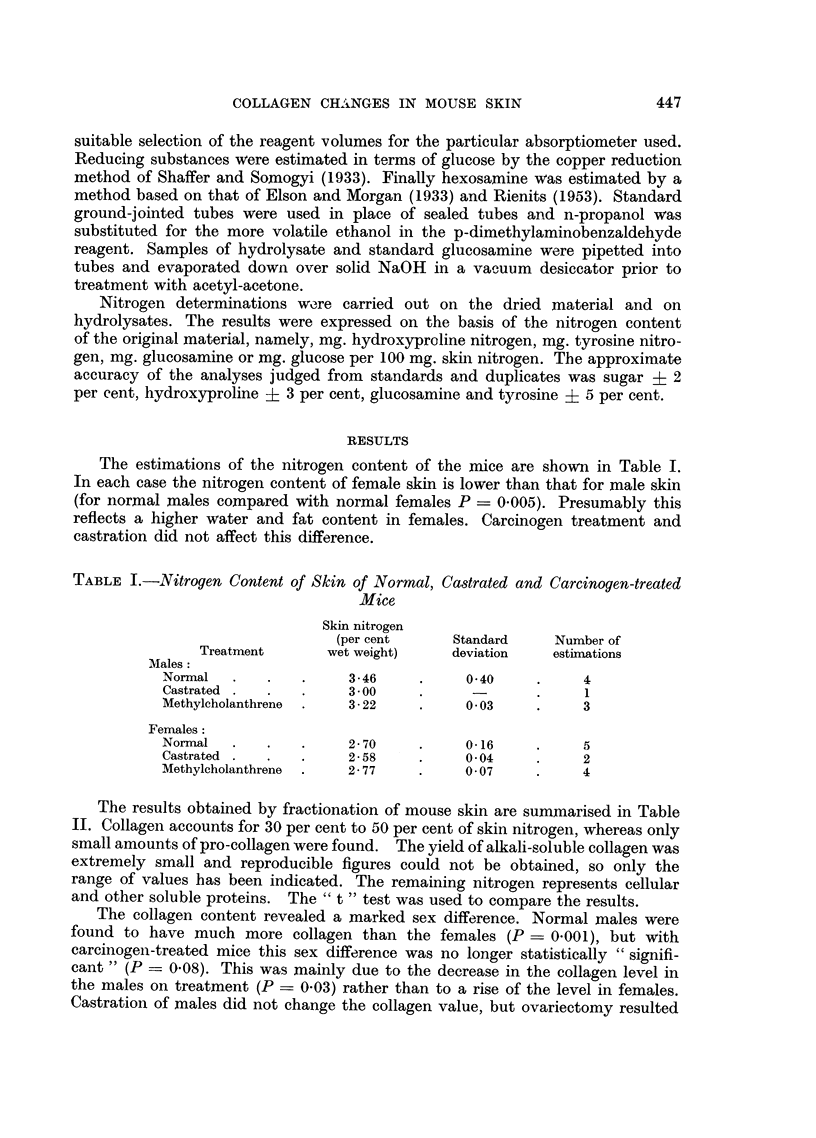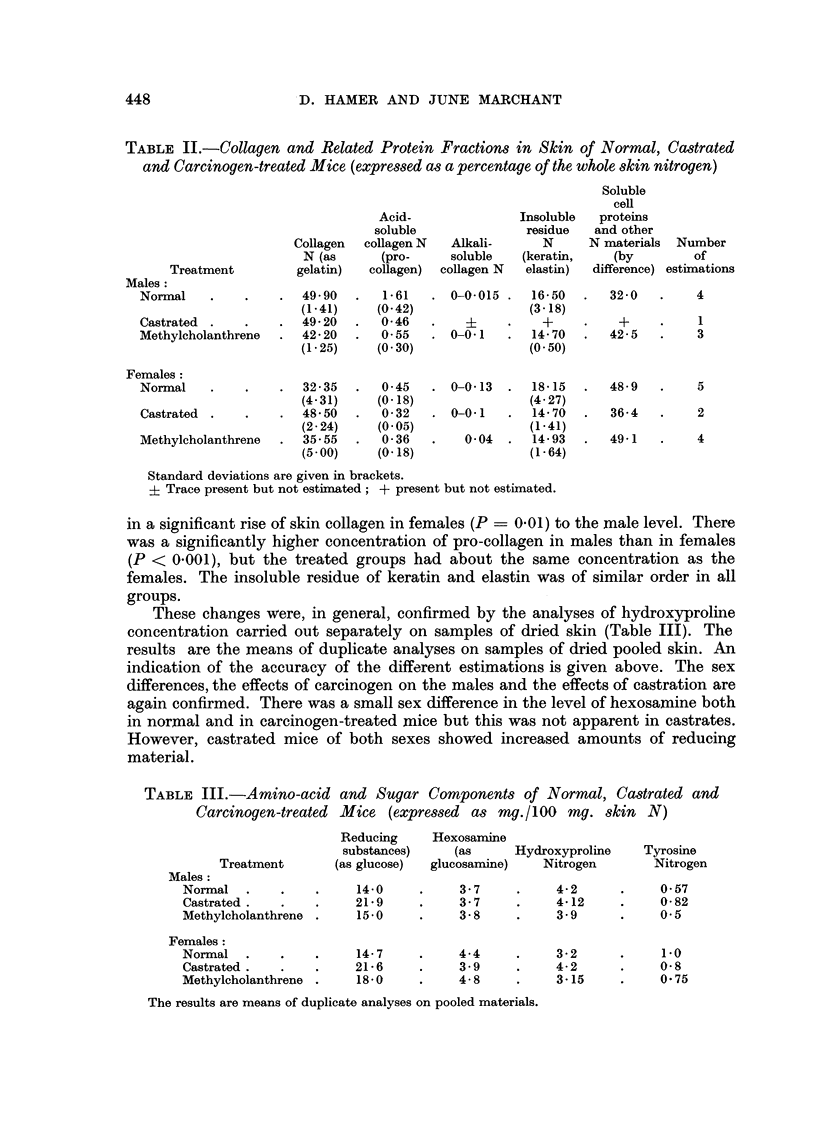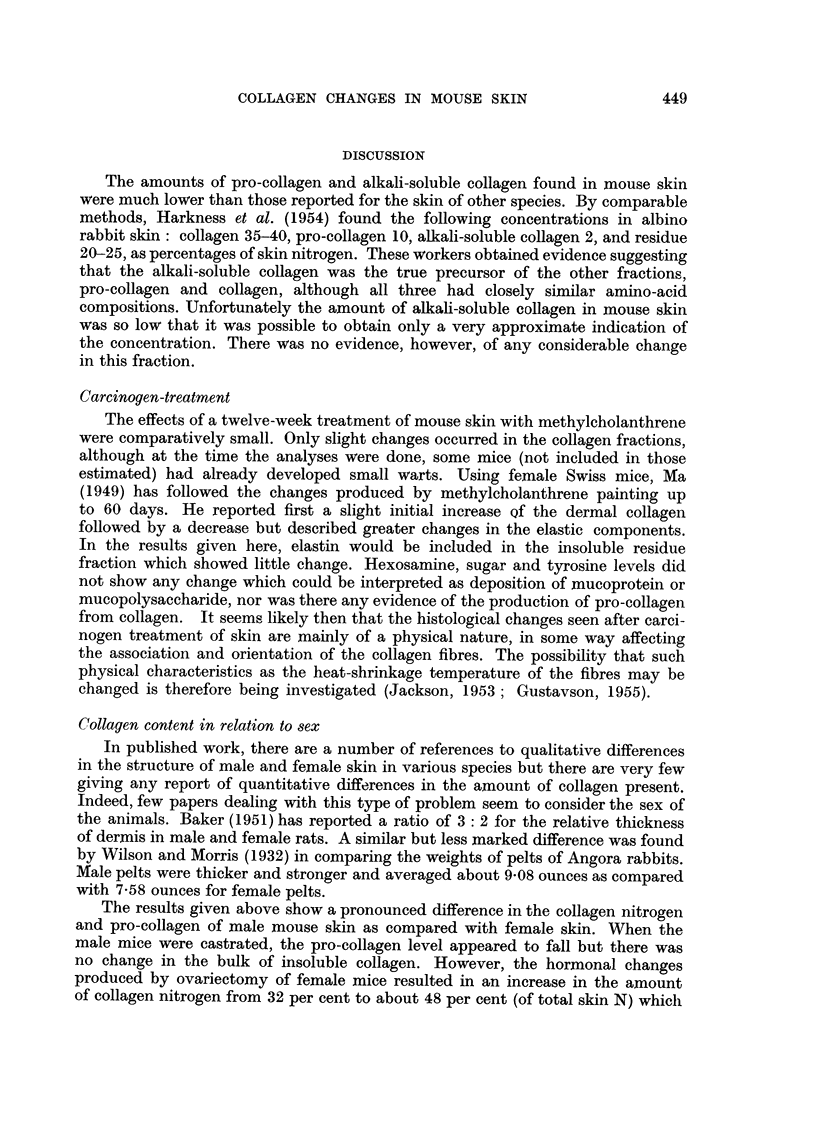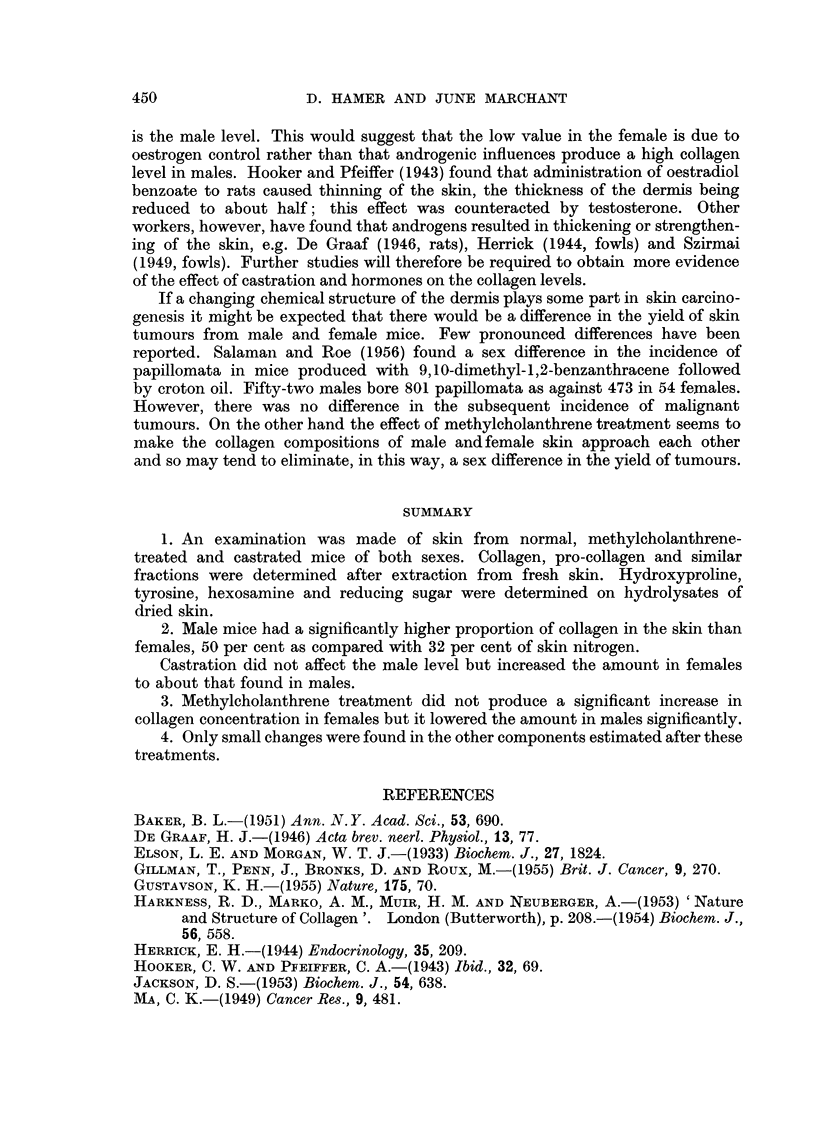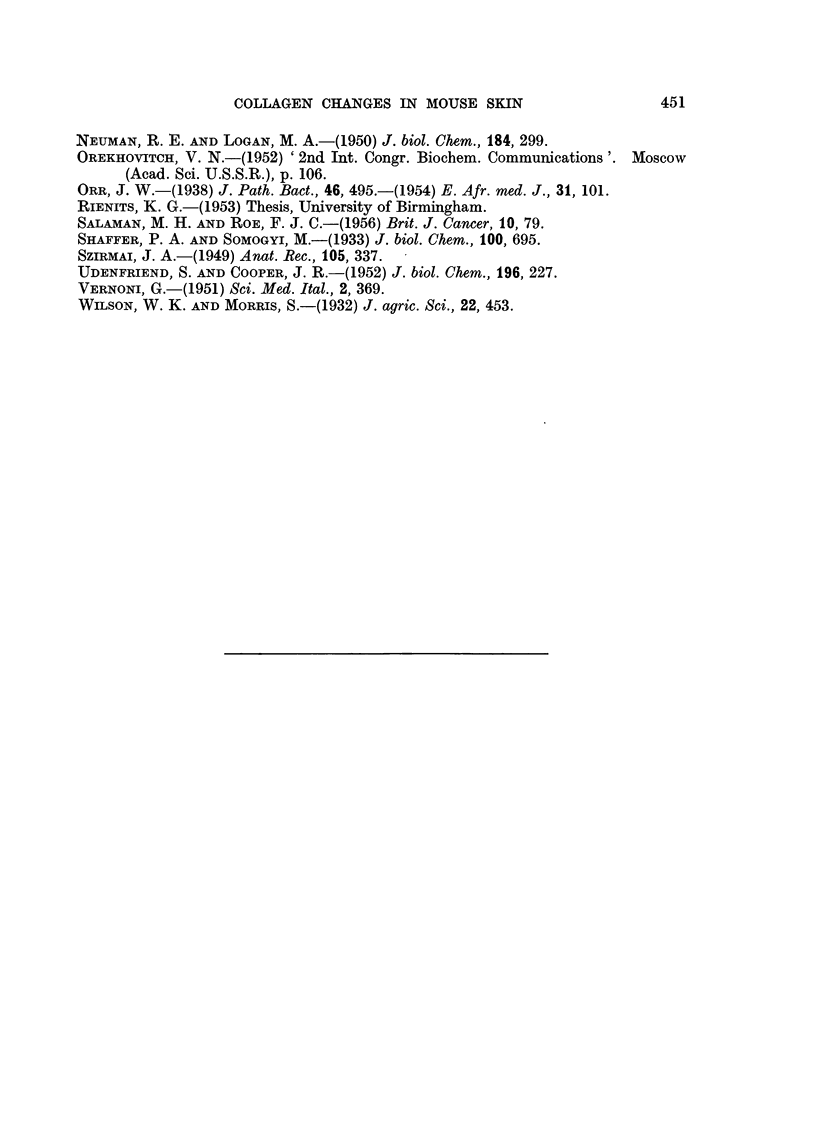# Collagen and Other Constituents in the Skin of Normal, Carcinogen-Treated and Castrated Mice

**DOI:** 10.1038/bjc.1957.52

**Published:** 1957-09

**Authors:** D. Hamer, June Marchant


### COLLAGEN AND OTHER CONSTITUENTS IN THE SKIN OF NORMAL, CARCINOGEN-TREATED AND CASTRATED MICE

D. HAMER* AND JUNE MARCHANT

*From the Cancer Research Laboratories, Department of Pathology, University of Birmingham*

Received for publication July 5, 1957

REPEATED application of carcinogenic hydrocarbons to mouse skin has been reported to result in changes in the structure of the dermis prior to the appearance of epidermal neoplasia (Orr, 1938 ; Vernoni, 1951). In normal mouse dermis the collagen consists of interwoven coarse bands of refractile fibres. During carcinogen treatment there is a progressive alteration beginning in the superficial dermis to fine, non-refractile fibres. This is sometimes associated with an increase in thickness of the dermis, suggesting there may also be formation of new collagen (Orr, 1938). Changes in the elastic fibres are also reported (Orr, 1938 ; Ma, 1949 ; Gillman, Penn, Bronks and Roux, 1955). It has been suggested that these stromal alterations may play an important part in the pathogenesis of mouse skin tumours. Similar changes have also been observed in human skin cancers (Vernoni, 1951) as well as in other human cancers (Orr, 1954).

In the work reported here an attempt was made to obtain more information about the cause of these histological changes by chemical studies on the connective tissue components in mouse skin during treatment with the carcinogen 20-methylcholanthrene. Collagen, certain acid-soluble (" procollagen ") fractions, polysaccharide and ground substance components were estimated in normal and treated skin. The results did not reveal any pronounced chemical changes and would suggest that physical changes in the state of association of the collagen, rather than changes in chemical composition, are responsible for the effects of carcinogens that have been observed histologically. A difference in the amount of collagen in male and female mouse skin was found and the effects of castration and ovariectomy were therefore examined.

### MATERIALS AND METHODS

### *Animals and treatment*

Outbred albino mice of both sexes were used. Water and cube diet (Thompson's formula) were available throughout. One group of mice was given weekly applications of 0·3 per cent (w/v) 20-methylcholanthrene in acetone for 12 weeks. The solution of the carcinogen was applied so as to cover most of the back (0·3 ml.). After the last painting the animals were left a further 2 or 3 weeks before being killed. The mice were then about 6 months old. The painted area of skin was shaved, removed and excess fat trimmed off. Skin was removed similarly from untreated control mice of similar age. The gonads were removed from another group of male and female mice about 10 weeks old which were kept a further 14

* Present address : Department of Chemistry and Pharmacy, College of Technology, Belfast.

weeks before being killed for skin analysis. Thus three groups of animals of either sex were used, i.e. normal, castrated and carcinogen-treated.

*Estimation of collagen and related proteins*

The general procedure followed was similar to that described by Harkness, Marko, Muir and Neuberger (1953, 1954). Pooled skin from 5 or 6 mice was minced finely with scissors and then further disintegrated for 2 minutes in a high speed mixer with 25 times its weight of cold, saturated $Na_2HPO_4$-solution. This and the subsequent operations were carried out so far as possible in the cold. This suspension was allowed to stand for 48 hours then centrifuged (Supernatant A). The residue was extracted with 0·1 *M* citrate-buffer (pH 3·8) for 24 hours and then centrifuged. The supernatant from centrifugation, on dialysis against 0·01 *M*-$Na_2HPO_4$, precipitated " acid-soluble collagen " or " pro-collagen " (Orekhovich, 1952). Material insoluble in phosphate and citrate was boiled under reflux with water for 24 hours to give a solution of gelatin derived from the fibrous collagen, and leaving an insoluble residue which contains proteins such as keratin and elastin.

The supernatant " A " from the original extraction was dialysed for 48 hours against water. The precipitate obtained was suspended in the citrate (pH 3·8) buffer for 24 hours and then centrifuged. The cell proteins in the sediment were discarded and the solution dialysed. " Alkali-soluble collagen " was then precipitated by dialysis against 0·01 *M*-$Na_2HPO_4$. (In fact, only extremely small amounts were isolated from mouse skin.)

*Estimation of nitrogen content of skin*

The nitrogen content of the original skin mince was estimated by micro-Kjeldahl digestion followed by colorimetric estimation of the ammonia with Nessler's reagent.

*Estimation of amino-acid and sugar components*

The following components were estimated : (*a*) hydroxyproline, as a measure of the collagen content, (*b*) tyrosine, representing other proteins and mucoproteins, (*c*) reducing materials, in terms of glucose, as a measure of polysaccharide and protein-bound carbohydrates and, finally, (*d*) hexosamine (as glucosamine)—a component of acidic mucopolysaccharides. Colorimetric methods were used in each case, the colour yield being determined against standards prepared at the same time and using an " EEL " photoelectric absorptiometer with the appropriate filter.

Pooled skin from several mice was dried *in vacuo* and then samples weighed out for hydrolysis. For the amino-acid estimations hydrolysis was carried out with 6 *N*-HCl for 18 hours at 107° C. in a sealed tube. The tubes were then opened and the solution washed out, filtered and taken to dryness on a steam bath before making up to a known volume. Hexosamine was estimated in specimens hydrolysed for 4 hours with 6 *N*-HCl in sealed tubes at 107° C. and reducing sugar in samples hydrolysed with 1 *N*-HCl for 4 hours under reflux.

The method of Neuman and Logan (1950) was used without modification for the estimation of hydroxyproline. Tyrosine was estimated with 1 nitroso-2-naphthol according to the description of Udenfriend and Cooper (1952) with suitable selection of the reagent volumes for the particular absorptiometer used. Reducing substances were estimated in terms of glucose by the copper reduction method of Shaffer and Somogyi (1933). Finally hexosamine was estimated by a method based on that of Elson and Morgan (1933) and Rienits (1953). Standard ground-jointed tubes were used in place of sealed tubes and n-propanol was substituted for the more volatile ethanol in the p-dimethylaminobenzaldehyde reagent. Samples of hydrolysate and standard glucosamine were pipetted into tubes and evaporated down over solid NaOH in a vacuum desiccator prior to treatment with acetyl-acetone.

Nitrogen determinations were carried out on the dried material and on hydrolysates. The results were expressed on the basis of the nitrogen content of the original material, namely, mg. hydroxyproline nitrogen, mg. tyrosine nitrogen, mg. glucosamine or mg. glucose per 100 mg. skin nitrogen. The approximate accuracy of the analyses judged from standards and duplicates was sugar $\pm$ 2 per cent, hydroxyproline $\pm$ 3 per cent, glucosamine and tyrosine $\pm$ 5 per cent.

### RESULTS

The estimations of the nitrogen content of the mice are shown in Table I. In each case the nitrogen content of female skin is lower than that for male skin (for normal males compared with normal females $P = 0{\cdot}005$). Presumably this reflects a higher water and fat content in females. Carcinogen treatment and castration did not affect this difference.

TABLE I.—*Nitrogen Content of Skin of Normal, Castrated and Carcinogen-treated Mice*

| Treatment | Skin nitrogen (per cent wet weight) | Standard deviation | Number of estimations |
|---|---|---|---|
| Males : | | | |
| Normal | 3·46 | 0·40 | 4 |
| Castrated | 3·00 | — | 1 |
| Methylcholanthrene | 3·22 | 0·03 | 3 |
| Females : | | | |
| Normal | 2·70 | 0·16 | 5 |
| Castrated | 2·58 | 0·04 | 2 |
| Methylcholanthrene | 2·77 | 0·07 | 4 |

The results obtained by fractionation of mouse skin are summarised in Table II. Collagen accounts for 30 per cent to 50 per cent of skin nitrogen, whereas only small amounts of pro-collagen were found. The yield of alkali-soluble collagen was extremely small and reproducible figures could not be obtained, so only the range of values has been indicated. The remaining nitrogen represents cellular and other soluble proteins. The " t " test was used to compare the results.

The collagen content revealed a marked sex difference. Normal males were found to have much more collagen than the females ($P = 0{\cdot}001$), but with carcinogen-treated mice this sex difference was no longer statistically " significant " ($P = 0{\cdot}08$). This was mainly due to the decrease in the collagen level in the males on treatment ($P = 0{\cdot}03$) rather than to a rise of the level in females. Castration of males did not change the collagen value, but ovariectomy resulted

TABLE II.—*Collagen and Related Protein Fractions in Skin of Normal, Castrated and Carcinogen-treated Mice (expressed as a percentage of the whole skin nitrogen)*

| Treatment | Collagen N (as gelatin) | Acid-soluble collagen N (pro-collagen) | Alkali-soluble collagen N | Insoluble residue N (keratin, elastin) | Soluble cell proteins and other N materials (by difference) | Number of estimations |
|---|---|---|---|---|---|---|
| Males : | | | | | | |
| Normal . . . | 49·90 | 1·61 | 0–0·015 | 16·50 | 32·0 | 4 |
| | (1·41) | (0·42) | | (3·18) | | |
| Castrated . . . | 49·20 | 0·46 | ± | + | + | 1 |
| Methylcholanthrene | 42·20 | 0·55 | 0–0·1 | 14·70 | 42·5 | 3 |
| | (1·25) | (0·30) | | (0·50) | | |
| Females : | | | | | | |
| Normal . . . | 32·35 | 0·45 | 0–0·13 | 18·15 | 48·9 | 5 |
| | (4·31) | (0·18) | | (4·27) | | |
| Castrated . . . | 48·50 | 0·32 | 0–0·1 | 14·70 | 36·4 | 2 |
| | (2·24) | (0·05) | | (1·41) | | |
| Methylcholanthrene | 35·55 | 0·36 | 0·04 | 14·93 | 49·1 | 4 |
| | (5·00) | (0·18) | | (1·64) | | |

Standard deviations are given in brackets.
± Trace present but not estimated ; + present but not estimated.

in a significant rise of skin collagen in females ($P = 0{\cdot}01$) to the male level. There was a significantly higher concentration of pro-collagen in males than in females ($P < 0{\cdot}001$), but the treated groups had about the same concentration as the females. The insoluble residue of keratin and elastin was of similar order in all groups.

These changes were, in general, confirmed by the analyses of hydroxyproline concentration carried out separately on samples of dried skin (Table III). The results are the means of duplicate analyses on samples of dried pooled skin. An indication of the accuracy of the different estimations is given above. The sex differences, the effects of carcinogen on the males and the effects of castration are again confirmed. There was a small sex difference in the level of hexosamine both in normal and in carcinogen-treated mice but this was not apparent in castrates. However, castrated mice of both sexes showed increased amounts of reducing material.

TABLE III.—*Amino-acid and Sugar Components of Normal, Castrated and Carcinogen-treated Mice (expressed as mg./100 mg. skin N)*

| Treatment | Reducing substances) (as glucose) | Hexosamine (as glucosamine) | Hydroxyproline Nitrogen | Tyrosine Nitrogen |
|---|---|---|---|---|
| Males : | | | | |
| Normal . . . | 14·0 | 3·7 | 4·2 | 0·57 |
| Castrated . . . | 21·9 | 3·7 | 4·12 | 0·82 |
| Methylcholanthrene | 15·0 | 3·8 | 3·9 | 0·5 |
| Females : | | | | |
| Normal . . . | 14·7 | 4·4 | 3·2 | 1·0 |
| Castrated . . . | 21·6 | 3·9 | 4·2 | 0·8 |
| Methylcholanthrene | 18·0 | 4·8 | 3·15 | 0·75 |

The results are means of duplicate analyses on pooled materials.

### DISCUSSION

The amounts of pro-collagen and alkali-soluble collagen found in mouse skin were much lower than those reported for the skin of other species. By comparable methods, Harkness *et al.* (1954) found the following concentrations in albino rabbit skin : collagen 35–40, pro-collagen 10, alkali-soluble collagen 2, and residue 20–25, as percentages of skin nitrogen. These workers obtained evidence suggesting that the alkali-soluble collagen was the true precursor of the other fractions, pro-collagen and collagen, although all three had closely similar amino-acid compositions. Unfortunately the amount of alkali-soluble collagen in mouse skin was so low that it was possible to obtain only a very approximate indication of the concentration. There was no evidence, however, of any considerable change in this fraction.

### *Carcinogen-treatment*

The effects of a twelve-week treatment of mouse skin with methylcholanthrene were comparatively small. Only slight changes occurred in the collagen fractions, although at the time the analyses were done, some mice (not included in those estimated) had already developed small warts. Using female Swiss mice, Ma (1949) has followed the changes produced by methylcholanthrene painting up to 60 days. He reported first a slight initial increase of the dermal collagen followed by a decrease but described greater changes in the elastic components. In the results given here, elastin would be included in the insoluble residue fraction which showed little change. Hexosamine, sugar and tyrosine levels did not show any change which could be interpreted as deposition of mucoprotein or mucopolysaccharide, nor was there any evidence of the production of pro-collagen from collagen. It seems likely then that the histological changes seen after carcinogen treatment of skin are mainly of a physical nature, in some way affecting the association and orientation of the collagen fibres. The possibility that such physical characteristics as the heat-shrinkage temperature of the fibres may be changed is therefore being investigated (Jackson, 1953 ; Gustavson, 1955).

### *Collagen content in relation to sex*

In published work, there are a number of references to qualitative differences in the structure of male and female skin in various species but there are very few giving any report of quantitative differences in the amount of collagen present. Indeed, few papers dealing with this type of problem seem to consider the sex of the animals. Baker (1951) has reported a ratio of 3 : 2 for the relative thickness of dermis in male and female rats. A similar but less marked difference was found by Wilson and Morris (1932) in comparing the weights of pelts of Angora rabbits. Male pelts were thicker and stronger and averaged about 9·08 ounces as compared with 7·58 ounces for female pelts.

The results given above show a pronounced difference in the collagen nitrogen and pro-collagen of male mouse skin as compared with female skin. When the male mice were castrated, the pro-collagen level appeared to fall but there was no change in the bulk of insoluble collagen. However, the hormonal changes produced by ovariectomy of female mice resulted in an increase in the amount of collagen nitrogen from 32 per cent to about 48 per cent (of total skin N) which is the male level. This would suggest that the low value in the female is due to oestrogen control rather than that androgenic influences produce a high collagen level in males. Hooker and Pfeiffer (1943) found that administration of oestradiol benzoate to rats caused thinning of the skin, the thickness of the dermis being reduced to about half; this effect was counteracted by testosterone. Other workers, however, have found that androgens resulted in thickening or strengthening of the skin, e.g. De Graaf (1946, rats), Herrick (1944, fowls) and Szirmai (1949, fowls). Further studies will therefore be required to obtain more evidence of the effect of castration and hormones on the collagen levels.

If a changing chemical structure of the dermis plays some part in skin carcinogenesis it might be expected that there would be a difference in the yield of skin tumours from male and female mice. Few pronounced differences have been reported. Salaman and Roe (1956) found a sex difference in the incidence of papillomata in mice produced with 9,10-dimethyl-1,2-benzanthracene followed by croton oil. Fifty-two males bore 801 papillomata as against 473 in 54 females. However, there was no difference in the subsequent incidence of malignant tumours. On the other hand the effect of methylcholanthrene treatment seems to make the collagen compositions of male and female skin approach each other and so may tend to eliminate, in this way, a sex difference in the yield of tumours.

### SUMMARY

1. An examination was made of skin from normal, methylcholanthrene-treated and castrated mice of both sexes. Collagen, pro-collagen and similar fractions were determined after extraction from fresh skin. Hydroxyproline, tyrosine, hexosamine and reducing sugar were determined on hydrolysates of dried skin.

2. Male mice had a significantly higher proportion of collagen in the skin than females, 50 per cent as compared with 32 per cent of skin nitrogen.

Castration did not affect the male level but increased the amount in females to about that found in males.

3. Methylcholanthrene treatment did not produce a significant increase in collagen concentration in females but it lowered the amount in males significantly.

4. Only small changes were found in the other components estimated after these treatments.

### REFERENCES

Baker, B. L.—(1951) *Ann. N.Y. Acad. Sci.*, **53**, 690.
De Graaf, H. J.—(1946) *Acta brev. neerl. Physiol.*, **13**, 77.
Elson, L. E. and Morgan, W. T. J.—(1933) *Biochem. J.*, **27**, 1824.
Gillman, T., Penn, J., Bronks, D. and Roux, M.—(1955) *Brit. J. Cancer*, **9**, 270.
Gustavson, K. H.—(1955) *Nature*, **175**, 70.
Harkness, R. D., Marko, A. M., Muir, H. M. and Neuberger, A.—(1953) 'Nature and Structure of Collagen'. London (Butterworth), p. 208.—(1954) *Biochem. J.*, **56**, 558.
Herrick, E. H.—(1944) *Endocrinology*, **35**, 209.
Hooker, C. W. and Pfeiffer, C. A.—(1943) *Ibid.*, **32**, 69.
Jackson, D. S.—(1953) *Biochem. J.*, **54**, 638.
Ma, C. K.—(1949) *Cancer Res.*, **9**, 481.

NEUMAN, R. E. AND LOGAN, M. A.—(1950) *J. biol. Chem.*, **184**, 299.
OREKHOVITCH, V. N.—(1952) ' 2nd Int. Congr. Biochem. Communications '. Moscow (Acad. Sci. U.S.S.R.), p. 106.
ORR, J. W.—(1938) *J. Path. Bact.*, **46**, 495.—(1954) *E. Afr. med. J.*, **31**, 101.
RIENITS, K. G.—(1953) Thesis, University of Birmingham.
SALAMAN, M. H. AND ROE, F. J. C.—(1956) *Brit. J. Cancer*, **10**, 79.
SHAFFER, P. A. AND SOMOGYI, M.—(1933) *J. biol. Chem.*, **100**, 695.
SZIRMAI, J. A.—(1949) *Anat. Rec.*, **105**, 337.
UDENFRIEND, S. AND COOPER, J. R.—(1952) *J. biol. Chem.*, **196**, 227.
VERNONI, G.—(1951) *Sci. Med. Ital.*, **2**, 369.
WILSON, W. K. AND MORRIS, S.—(1932) *J. agric. Sci.*, **22**, 453.